# Dietary Fiber: Fractionation, Characterization and Potential Sources from Defatted Oilseeds

**DOI:** 10.3390/foods10040754

**Published:** 2021-04-02

**Authors:** Gita Addelia Nevara, Sharifah Kharidah Syed Muhammad, Norhasnida Zawawi, Nor Afizah Mustapha, Roselina Karim

**Affiliations:** 1 Department of Food Science, Faculty of Food Science and Technology, Universiti Putra Malaysia, Serdang 43400, Selangor, Malaysia; gitanevara@yahoo.co.id (G.A.N.); kharidah@upm.edu.my (S.K.S.M.); norhasnida@upm.edu.my (N.Z.); 2 Department of Nutrition, Universitas Mohammad Natsir Bukittinggi, Sumatera Barat 26100, Indonesia; 3 Department of Food Technology, Faculty of Food Science and Technology, Universiti Putra Malaysia, Serdang 43400, Selangor, Malaysia; nor_afizah@upm.edu.my

**Keywords:** dietary fiber, fractionation, functional, oilseed by-product, rheological

## Abstract

Dietary fiber (DF) has wide applications, especially in the food and pharmaceutical industries due to its health-promoting effects and potential techno-functional properties in developing functional food products. There is a growing interest in studies related to DF; nevertheless, there is less focus on the fractionation and characterization of DF. The characteristics of DF fractions explain their functionality in food products and provide clues to their physiological effects in food and pharmaceutical industrial applications. The review focuses on a brief introduction to DF and methods for its fractionation. It discusses the characterization of DF in terms of structural, physicochemical and rheological properties. The potential sources of DF from selected defatted oilseeds for future studies are highlighted.

## 1. Introduction

Dietary fiber (DF) is an essential nutrient that is resistant to the digestive enzymes in the small intestine. However, it can be partially or fully fermented in the large bowel [1] Fractionation of DF aims to isolate and quantify fractions and eliminate undesirable compounds. The relative number of individual fiber constituents, especially in relation to soluble and insoluble fractions, affects the physicochemical and physiological attributes of DF [2]. 

A study on the structural characterization of polysaccharides is necessary to provide a better understanding of their function as DF. The different methods used in the fractionation resulted in different structural characteristics of the compound. Moreover, DF has essential functional properties such as water- and oil-holding capacity, emulsification and gel formation, and rheological properties that are required in developing novel food products [3]. These properties may explain its role in food products and provide clues to its physiological effects when extended to industrial applications. Furthermore, analyzing the rheological behavior of DF is crucial specifically in food product development, storage stability, sensory evaluation, quality control, food structure and design of food processing equipment [2,3].

Even though there is a growing number of studies on DF, limited literature about the fractionation and characterization of DF, and potential sources of DF from defatted oilseeds are available. The fractionation of DFs into their constituents with specific physical characteristics and chemical contents may improve their functionality. Furthermore, the utilization of the by-products of oilseeds such as oilseed meal or cake into high value-added food ingredients with health-promoting properties will benefit mankind. Therefore, this review focuses on a brief introduction to DF and its fractionation methods, and elaboration of the characteristics of DF fractions in terms of structural, functional, and rheological aspects. It also provides information on some potential defatted oilseeds as a source of DF.

## 2. Fractionation of Dietary Fiber (DF)

### 2.1. Introduction of DF

Eben Hipsley was the first person to use the term dietary fiber (DF) and in 1953, he observed that people with diets high in fiber-rich foods tended to have lower pregnancy toxemia levels [1]. Previously the analytical term “crude fiber” was used to denote the portion of plant foods that escaped solvent, alkali, and acid extractions [4]. These terms have been used interchangeably, but DF is defined as carbohydrate complex which provide the rigid structure of plant cell wall [5] and escape digestion and absorption in the upper human gastrointestinal tract (GIT) [6], while crude fiber is the remaining part of DF (mainly lignin and cellulose) after being treated with acid and alkali [7]. 

DF refers to a chemical complex that can react and interact within the food matrix and the human digestive system [2]. The intestine can be directly affected by DF through the alteration of digestion and absorption patterns [3]. DF consists of insoluble and soluble forms that vary in physiological and physicochemical attributes [8]. Soluble DF is characterized by its water solubility and viscosity, which lowers the blood cholesterol and triacylglyceride concentrations modestly and attenuates the postprandial glucose response. Insoluble DF is characterized by porosity and density and its capacity in increasing fecal mass and decreasing intestinal transit time, thus enhancing intestinal peristalsis [9]. The mechanism for DF postprandial hyperglycemia reduction is the direct delaying effect on the absorption of glucose in the GIT due to a modification in the diffusion of the final product digestion within the lumen [10]. Thus, viscous DF forms can alter events (such as glucose absorption rate) occurring within the GIT [8].

DF represents a wide range of carbohydrate components with different structures that escape digestion and absorption within the upper GIT part [11]. High-fiber diets also help fecal bulking and decreased transit time, thus reducing postprandial glycemic response, regular blood cholesterol maintenance, and lowering the risk of developing coronary heart disease [12]. These positive impacts are due to the non-starch polysaccharides comprising the plant cell walls. Therefore, it is essential to study the composition and physicochemical attributes of the DF fraction [13].

### 2.2. Fractionation of DF

Fractionation of DF can be conducted using dry or wet processes to isolate starch and protein, and a fiber fraction is obtained as an end product [14]. There are several fractionation processes, differing by the method applied, separation techniques, and pretreatment practices. The parameters, such as the cost, time, yield, technological characteristics, and the functionality lost during the fractionation, change considerably according to the fractionation process applied [15]. Fractionation of DF isolates the interested fractions, quantify those constituents, and eliminate unfavorable components. There are limited methods for the fractionation of DF into their constituents. It is recognized that the physicochemical and physiological effects of DF depend on its individual components, especially in relation to insoluble and soluble fractions [16].

Southgate [17] was the first to fractionate the unavailable carbohydrates in foods, which include the extraction and fractionation procedure for crude lignin, cellulose, and lignocellulose fractions [18]. Also, wheat bran was fractionated using a hot and cold water extraction to isolate the water-soluble polymers and enzymatic and acid treatments to fractionate the insoluble fibers [19]. Furthermore, combined fractionation methodologies using heat resulted in the modified insoluble fiber fraction levels [20]. Graham et al. [21] found that high-temperature extraction contributed to the highest yield of soluble fibers, and acidic extraction yielded the lowest. Czuchajowska and Pomeranz [22] patented the wet fractionation method to isolate starch, protein, and DF, requiring no chemicals and much less water than other standard methods. DF is a significant component of both water-soluble and tailings starch fractions and large amounts of protein and starch [23].

Alternatively, Wang et al. [24] employed a dry fractionation that is water- and energy-efficient and does not need any solvents to produce enriched DF from defatted rice bran. Also, the dry fractionation technique creates fractions with different particle sizes and densities that affect their fiber content [25]. Yáñez et al. [26] applied dry fractionation on distillers dried grains with solubles (DDGS) using a vibratory sifter and gravity separator and found that this technique was more effective than wet fractionation due to its cost-effective, environmental-friendly method and high yield. Therefore, dry fractionation could be conducted as a tail-end method at ethanol plants to separate DDGS into fragments [27].

The various fractionation methods are developed based on the material evaluated; thus, a global fractionation procedure is unavailable [16]. The aforementioned techniques only describe universal fractionation methods. Hence, each researcher should modify previous procedures to develop an optimum method for a specific sample [16]. Several methods enable a more refined separation of constituents, allowing the evaluation of molecular structure, e.g., pectin [28]. Following the extraction, isolation, and purification using chromatographic techniques, the molecular weight of polysaccharides can be evaluated by high-performance liquid chromatography (HPLC), and the structure is confirmed by nuclear magnetic resonance (NMR) [29]. Recently, Alba et al. [30] developed a sequential fractionation procedure of blackcurrant pomace into five insoluble and soluble DF fractions. In commercial applications, dry fractionation uses pin milling and air classification, which is repeated to obtain a high recovery level of the protein fraction [14]. The efficiency of milling and air classification varies considerably due to differences in structural thickness and hardness of cell walls and seeds and binding strength between starch granules and protein [31]. 

The variation in starch, protein, and minor component levels in the fractions will influence functionality [14], thus, affecting the overall product quality produced from the fraction. Food product development can be successfully achieved by understanding the particular functional attributes of the constituents and their performance under different treatments such as temperature and pH [32,33].

### 2.3. Characterization of DF

There is a considerable variation in the DF amounts and insoluble to soluble DF ratios [34]. The characteristics of plant varieties are required to interpret the physiological function of the fibers better. There are several types of DF, including long-chain insoluble and soluble polysaccharides, galactooligosaccharides, and resistant starch. While insoluble DF is commonly associated with laxation, soluble DF reduces cholesterol levels and ameliorates postprandial blood glucose levels. All DF can serve as prebiotics, which provides food for gut microbiota [13,35].

The efficacy of DF in promoting health benefits depends on its intake, source, and structural and chemical composition. Moreover, a substantial understanding of the chemical structure of DF is required when incorporating DF into food products as DF will interact with other ingredients that can remarkably modify the microstructure and characteristics of the final food product [30]. The basic composition of DF has been determined; however, the study on the full characterization of the non-starch polysaccharides is limited. This knowledge is important for learning the effects of structure on the functionality of these DFs and how the physicochemical properties of DF fractions can affect the final processed foods [34].

The characteristics of the cell wall polysaccharides in cotyledons and seed hulls are essential for understanding their function as DF. The forms of sugars exist and the physical properties of materials are less important than the linkage of constituent monosaccharides in polysaccharides [36]; different monosaccharides linked in the same manner can give similar physical attributes to materials. In contrast, the same monosaccharide linked in different manners can provide polysaccharides with completely different attributes [34].

The profiles of small molecular weight carbohydrates i.e., galactooligosaccharides of cooked seeds are also of interest. These molecules were previously considered undesirable due to their flatulence effect [13]. However, there is increasing recognition of their prebiotic effect, which stimulates the growth of probiotic bacteria to produce beneficial short-chain fatty acids [33].

For the carbohydrate characterization, resonances 1H NMR and 13C NMR are the most appropriate spectra for analyzing monosaccharides [37]. In this regard, the 1HNMR (<1 ppm) detects CH3-groups, while the 1HNMR (>2 ppm) are suitable to detect *O*-acetyl and *N*-acetyl groups [38]. NMR spectroscopy is a potent analytical method for analyzing the structure, type, and several glycosidic linkages of carbohydrates and α- and β-anomeric configurations in the molecules [39]. NMR is considered as a non-destructive rapid technique to obtain the structural information of molecules [37]. For example, the chemical structure of multiple carbohydrates such as macroalgae gums (i.e., carrageenan and alginates) has recently been analyzed using NMR methods [40].

The soluble and insoluble nature of DF involves variations in their technological functionality and physiological properties [41,42]. Soluble DFs are characterized by their ability to increase the viscosity and decrease glycemic response and plasma cholesterol [42,43]. Insoluble DFs are characterized by their low density, porosity, and capacity to increase fecal bulk and reduce intestinal transit [42,44]. Compared with insoluble DF, the soluble fraction exhibits a better capacity to form gels, provide viscosity, act as emulsifiers, has neither unpleasant taste nor undesirable texture, and is simpler to incorporate into convenience food and beverage. Fruit by-products and marine algae seem to be excellent sources of soluble DFs, followed by vegetables, fruit, and cereals [16].

## 3. Structural Characterization of DF

Due to variability in structures of polysaccharides, some methods are used to characterize their morphological structures. Determining the distribution of ingredients with various physical and chemical characteristics will give another insight. The structural characterization of DF involves a determination of monosaccharide composition, molecular weight, Fourier-transform infrared spectroscopy (FTIR), X-ray diffraction (XRD), and scanning electron microscopy (SEM). Table 1 shows the structural characterization of DF from various sources.

### 3.1. Monosaccharide Composition

Besides water, carbohydrates are the most common food and human dietary components (natural and added ingredients). Carbohydrates are essential as energy sources and ingredients that impart texture, and as DF that contribute to human health [57]. Carbohydrates have various sizes and shapes, from simple sugar (saccharide) units to complex polymers consisting of thousands of simple sugar molecules [58]. 

Monosaccharides are carbohydrate molecules that cannot be hydrolyzed into smaller units. Therefore, monosaccharides are known as “simple sugars”, which refers to the simplest carbohydrates [57]. A monosaccharide is the only carbohydrate molecule that can be absorbed from the small intestine [59]. Monosaccharide means one sugar, demonstrating a molecule composed of only one sugar unit and not two or more units of sugar combined [57].

Oligo- and polysaccharides which are the higher saccharides must be hydrolyzed to monosaccharides before it is absorbed and utilized [59]. Two of the essential monosaccharides in foods are six-carbon sugars i.e., fructose, and glucose with the general formula C_6_H_12_O_6_ [58]. HPLC is the technique for analyzing mono- and oligosaccharides in food samples and also for polysaccharides that have been hydrolyzed into their constituent monosaccharides [59].

### 3.2. Molecular Weight

There is growing evidence that the molecular weight and structural features of polysaccharides are of fundamental significance since they are associated with the physicochemical and functional properties of polysaccharides [60]. For example, the high molecular weight and typical molecular structure of corn fiber gum are responsible for its good emulsifying properties [61]. 

Since all polysaccharide preparations are polydisperse, the average molecular weight information is needed [62]. The carbohydrates can be separated based on the molecular weight of compounds by size-exclusion chromatography (SEC) [37].

The rapid analysis of molecular weight range and average molecular weight of polysaccharides (amylopectin, amylose), soluble gums (guar, xanthan, pullulan), and cellulose derivatives can be carried out using hydrophilic polymeric size-exclusion packings [59]. Molecular weight distribution can be analyzed directly from high-performance SEC if multi-angle laser light scattering (MALLS) or low-angle laser light scattering (LALLS) are employed for detection [63].

The SEC test is helpful to figure out numerous food compositions and systems. SEC investigation of tomato cell wall pectin from hot- and cold-break tomato processing demonstrated that the different processing procedures did not differentially degrade the cell wall pectin [59]. Size-exclusion HPLC is a rapid, straightforward technique for determining soybean cultivars based on protein content. This method has also been applied to determine polymerized triacylglycerols in fats and oils [63].

### 3.3. Fourier-Transform Infrared Spectroscopy

Fourier-transform infrared (FTIR) spectroscopy is a powerful method of screening foods due to its fast, easy, and cost-effective method. It can also provide data about the origin of specific foods [64]. The chemical bonds can be identified by FTIR spectroscopy analysis using an infrared spectrum absorbed by the sample [65]. Spectroscopy is a powerful non-destructive method that employs an electromagnetic radiation interaction effect to evaluate the molecular structure or atomic and the energy level of the material [66]. FTIR tools utilize the interference between two IR beams to produce a signal, known as an interferogram, which is a function of the change in path length between the two beams [67] using a Michelson interferometer [68]. Solid foods can be analyzed by FTIR using alkali halide discs. These discs are made from potassium bromide (KBr) [64], and are apparently the preferred method in several researches, such as detecting fraud in saffron samples [69] and roasted coffee [70]. However, KBr discs do not provide the desired reproducibility for identifying food adulteration or establishing authenticity; thus, the attenuated total reflectance (ATR) FTIR is used as an alternative method due to the higher reproducibility and minimal sample preparation [64].

### 3.4. X-ray Diffraction

X-rays can be used for quality checking through diffraction, imaging, or scattering modes [71]. Diffraction methods can identify different compounds with the same composition because it detects the crystalline structure, not the chemical compositions [72]. Diffraction methods include X-ray diffraction (XRD), neutron diffraction, and electron diffraction. XRD is the most effective technique for identifying the crystal structure of materials [71]. XRD was invented in 1912 and has been the most extensively studied and used method for materials characterization [72]. Moreover, XRD is broadly applied to analyze changes in the crystallinity and structure of cereal starches after various processing methods [71].

XRD techniques consist of two types: spectroscopic and photographic. The spectroscopic method (standard XRD method) is the most extensively used diffraction technique in modern laboratories compared to photographic techniques because spectroscopic techniques can replace most photographic approaches [72]. XRD data can interpret the crystal polymorphism and the fraction of amorphous and crystalline content. Various software applications are available to simulate diffraction data, such as X’Pert High Score Plus, or Crystal Diffract [71]. The XRD has an excellent capability and plays a progressively significant role in the structural characterization of natural products due to the possibility to provide absolute structure determination, structure determination in the presence of solvents in the crystal unit, and packing of molecules in the crystal [73].

### 3.5. Scanning Electron Microscopy

A study on structural properties is a means to analyze pore formation and distribution instead of the measurement of an average value of a property for the whole food component [74]. Electron microscopy (EM) is an advanced method to reveal the microstructure and the pore distribution, which employs electrons as the imaging medium. EM consists of transmission electron microscopy (TEM) and scanning electron microscopy (SEM) [59].

SEM examines microstructure by scanning the surface of substances, similar to scanning confocal microscopes but with much greater field depth and much higher resolution [75]. SEM employs an electron beam to scan across the surface of a specimen. After interacting with the materials, electrons come out in various forms [59]. Perhaps the most crucial feature of an SEM is the three-dimensional appearance because of its considerable field depth [75].

Investigation of surface morphology using SEM can elucidate the network properties and microstructure of a combination. SEM has been employed in the investigation of the structural properties of freeze-dried rice as influenced by process conditions [76], the effects of xanthan, locust bean, and guar on sucrose [77], and tapioca starch modified by xanthan and guar gum [78]. 

## 4. Functional and Physicochemical Characterization of DF

The health benefits and physiological effects of DF are challenging to predict based on their structural characteristics alone; however, they can be predicted based on physicochemical and functional properties [79]. Thus, the physiological properties of DF depend on a complex combination of physical, chemical, and structural attributes [80]. Determination of functional properties of DFs such as water- and oil-holding, swelling and gel-forming capacities from various DF sources are shown in Table 2.

### 4.1. Water-Holding Capacity

The amount of water contained in a sample when equilibrated at a set water activity is called water-holding capacity (WHC) [59]. WHC refers to the capacity of a sample to hold water, and it is defined as the moisture content of the sample after reaching equilibrium under certain conditions [83]. This parameter calculation is fundamental for storage condition determination and investigation of cost-effectiveness for food formulations, which alters the textural, stability, and sensory characteristics of food and pharmaceutical products [84]. Furthermore, the study has shown that the milling technique influences the particle size distribution and other flour properties, including water-holding capacity [85].

### 4.2. Water-Solubility

The hydration and solubility seem to be the same, but each has a different description. Hydration refers to the reaction of water with a soluble substance, but the solubility defines the soluble material in the solvent. Solubility represents the powder behavior in an aqueous solution [83]. Various aspects such as the drying method, solute structure, nature of the solvent, wettability, and processing conditions are involved in the powder solubility [86]. Among these aspects, the drying method significantly affects the powder composition (such as protein and monosaccharides), the molecular weight of components and chemical conformation [83]. The studies showed that the oven-dried powders were less soluble than spray and freeze-dried powders [87].

### 4.3. Water-Retention Capacity

A detailed characterization of the hydration attributes of the sample requires a combination of certain techniques as the different techniques change the hydrated states, hence resulting in different values of hydration properties [88]. Water-retention capacity (WRC) determines the water retained by the insoluble matrix after applying an additional force and is calculated as the amount of water retained per g of dry sample residue [34]. The WRC of fruit or vegetables is changed once cooked because the cell membranes lose their selective permeability and allow additional nutrients, sugars and water. When substances move from higher to lower concentration, diffusion occurs, and the plant cell loses its water, form, and turgor [58]. For the hydration properties, “Profiber” definitions and protocols were elaborated by Robertson et al. [89], and pea hulls were used as one of the fiber substrates in developing the measurement protocols which was undertaken in two stages.

### 4.4. Water-Swelling Capacity

The swelling capacity refers to the ratio of the volume occupied by the wetted material after equilibration to the actual sample weight [90]. For example, 200 mg of fiber is hydrated with 10 mL of water for 18 hours, without external stress except for gravity [34]. The swelling capacity is one of the physicochemical properties that affect the technological and physiological attributes of DF in food systems [91]. It was suggested that the swelling capacity of soluble DF in functional foods was positively associated with the rheological attributes of final products and the palatability experienced by consumers [92].

### 4.5. Oil-Holding Capacity

Oil-holding capacity (OHC) is the ability of a sample to absorb oil under specific test conditions, similar to that method to estimate water-holding capacity [93]. OHC values of DF reflect its density, surface properties, polysaccharide structure, and other aspects related to the extraction process [16,94]. Incorporating the DF-rich ingredients produces food products with lower calorie, fat, and cholesterol level [93]. They can also act as functional ingredients improving viscosity, hydration, OHC, sensory quality, texture, and shelf-life [16].

### 4.6. Viscosity

The utilization of DF as a functional substance in the production of novel products enhances structural and physical attributes, including viscosity [16]. Water-soluble DFs are the primary constituent that would increase the viscosity of a solution [43]. The mechanisms of the viscosity increment by DF depend mainly on the physical properties of the DF rather than its chemical composition [34]. In contrast, the susceptibility of the colon to fermentation depends on the chemical structure [36].

### 4.7. Gel-Forming Capacity

Gels refer to the viscoelastic ingredients, which possess characteristics ranging from liquids to solids, depending on the experimental period [93]. The food industry prefers soluble DF over insoluble DF due to gel formation and emulsification and as an ingredient in foods and beverages without affecting taste [16]. DF can be employed as thickeners in several products such as jellies, jams, or marmalades and also help in avoiding syneresis in the products, maintaining texture stability and gel formation, and improving product quality [93]. For example, DF from peach was used in jams to provide the required functional property [95].

### 4.8. Antioxidant Capacity

Plant non-starch polysaccharides can be exploited as potential novel antioxidants due to their antioxidant contents [16]. Several antioxidant capacities are found in the polysaccharide fractions from rice bran, such as protection against the hydroxyl free radical, superoxide radical, lipid peroxidation and promising potential in reducing power and chelating ferrous ions [96]. The insoluble DF fraction exhibited antioxidant power about 20-fold higher than soluble DF or whole cereal, with ferulic acid being the highest (67–87%) phenolic compound [97]. The metabolites with considerable free radical scavenging capacity were produced by several compounds in their transit through the GIT creating an antioxidant ambiance to prevent cancer [98,99].

### 4.9. Water Activity (a_w_)

Food products with the same moisture content have different perishability as the moisture content alone does not represent a decent food stability indicator [100]. The water activity of foods correlates with physical and chemical properties, enzymatic reactions, and microbial growth [59]. Water activity (a_w_) refers to a thermodynamic characteristic of water in a food sample determined as the ratio of the fugacious trend of the water in the material to the fugacious of water at the same pressure and temperature [100,101,102]. The a_w_ of foods alters essential safety and quality aspects, and therefore a_w_ analysis is frequently used in food production [59].

## 5. Rheological Characterization of DF

### 5.1. Introduction of Rheology

Rheology is the branch of physics studying how materials flow or deform in response to applied mechanical stresses or forces [103]. It studies the mechanical characteristics of liquid flow and solid deformation as stress, strain, and time [104]. In particular, rheology concerns the behavior of materials in the transient area between solids and fluids [105]. The rheological properties refer to the attributes of material that govern the particular way these flow behaviors or deformation occur [103]. Rheological properties are crucial in designing, modeling, and evaluating processes and rheological data to determine process characteristics involving fluid flow, such as extraction, filtration, pump sizing, and purification [106]. 

In particular, rheological techniques accurately measure deformation, flow, and force and analysts must consider how best to address this information [59]. Flow behavior is one such response to deformation or force [107]. In physics, a continuous deformation over time is called “flow”, and all materials can flow. Thus, the capacity to flow is possessed by liquids and gases, and solids to a varying degree [103]. The rheological properties are composition, temperature, and other processing parameters, such as the fluid speed through a pipe. The influence of these parameters on flow properties can be measured by viscosity using a viscometer or rheometer [107]. 

Basic concepts of strain (deformation per length) and stress (force per area) are keys to all rheological determinations [105]. Stress (σ = applied force/area) resembles the magnitude of force components applied to a material object. While strain (γ = deformation/original size) is the difference in the size or shape of a material object due to the force applied, expressed as the ratio or percent change in the original size and shape; the strain is, therefore, a non-dimensional factor denoting movement [104]. It is essential to understand that, following the inherent rheological attributes of a sample, the amount of deformation detected in a material in response to a given applied force will also depend on the shape and geometric size of the material [103].

### 5.2. Food Rheology

Food rheology refers to material science devoted to foods [59]. Foods are very complex structured materials consisting of water, carbohydrates, fats, proteins, and fibers. All these components affect the structural behavior and flow of foods remarkably and, therefore, the rheological attributes of food are completely different from conventional polymeric materials [108]. Whether working in quality control, process design and scale-up, or product development, rheology has an integral role in producing high-quality food products [107]. For instance, the flow of salad dressing from a bottle and the snapping of a candy bar are related to the rheological properties of these materials [59]. Also, the rheological properties of hydrocolloids provide data for determining attributes to modify the texture of food products [104]. The stability of structured fluids is influenced by various influences. The viscosity of the liquid phase in dispersions mostly becomes a major factor in the flow properties of material [108]. Also, many aspects of food safety are affected by rheological characteristics. For example, the flow properties of liquid foods during continuous thermal processing are directly related to the amount of time the food is stored in the system (residence time), and the amount of heating (thermal dose) received [107].

Nowadays, food rheology is not only a measurement of apparent viscosity but also in-depth data on the fluidity and microstructure of food. A revolution from a rotational viscometry to either controlled stress/strain rheometer or a more developed Fourier transformation rheometer carried more accurate, reliable and sophisticated rheological data [108]. Furthermore, studying the rheological properties of food resulted in food processing equipment, sensory evaluation, quality control, storage stability, food structure, and food product development [106,109]. There is increasing attention to research related to food microstructure and its correlation with the rheological properties of food [108]. Also, controlling the rheological behaviors of particles in dispersion has been of primary interest in the industry [110]. The knowledge of food rheology and microstructure helps minimize textural defects in processed foods and accomplish consumer expectations [108].

### 5.3. Measurement of Rheological Properties 

In a sensory test of a food product, perceived consistency, taste, appearance, and juiciness are the main properties to be evaluated. Therefore, it is essential to determine and control the food rheological properties [111]. Semi-solid and fluid food products (e.g., mayonnaise, chocolate, peanut butter, ketchup) are regularly observed using steady and oscillatory rheological tests for quality maintenance [108].

Viscosity, “the resistance to flow”, is determined as shear stress to shear rate ratio [16]. Fluids can be classified as non-Newtonian and Newtonian. Viscosity applies to Newtonian fluids, and apparent viscosity applies to non-Newtonian fluids [104]. An increasing shear rate results in increasing or decreasing viscosity of polysaccharide fluids as most polysaccharide solutions reveal non-Newtonian flow [112]. Water-soluble DF is the primary constituent that plays a role in the viscosity increment of a solution [43,44]. Abdul-Hamid and Luan [43] found that defatted rice bran contains only 9% soluble DF has a low viscosity (approximately 1.25 cps at 7% fiber in water). An increased fiber concentration results in viscosity increment; however, it decreases with the temperature of a DF solution. Grigelmo-Miguel et al. [113] and Elleuch et al. [114] demonstrated that peach and date flesh DF suspensions showed pseudoplastic fluids, whose apparent viscosity decreases immediately with a shear rate increment, as defined by the power-law model (C = Kcn). The flow behavior index (n) determined the degree of pseudoplastic behavior [16]. For example, DF suspensions of date (between 20 and 50 g/L) shows pseudoplastic behavior (n < 1). This index passes from 0.326 to 0.13 with an increase in the concentration of DF suspensions from 20 to 50 g/L [114]. Viscosity is a property to be considered in processes that involve polymer solutions in which rheological behavior is complex [109]. The viscosity of polymer solution is more difficult to predict and correlate than the viscosity of systems containing low molecular weight compounds [106]. Commonly, the addition of hydrocolloids increases the viscosity and affects the rheological properties of solutions or pastes [115]. Table 3 shows the rheological measurement of DF in different sources. The extensional flow has industrial significance among various types of flow. Investigations of extensional viscosity of liquid foods are essential in process modeling and design, process and quality control, and structural characterization of products [108]. On the other hand, particle size is a crucial indicator that affects inherent food characteristics and the final food product quality [116].

## 6. Potential Sources of DF from Defatted Oilseeds

Defatted seed cakes are seed flours in which their fat content have been removed partially or fully, which subsequently improved the protein content of the resultant seed cake. The exploitation of by-products generated from food processing as a source of functional ingredients and their application in other foods is necessary as part of a waste management system [124]. The by-product of oilseeds such as defatted cakes from kenaf, hemp and sesame seeds are some of the potential low-cost sources for DF extraction. The number of nutritional contents of these by-products is shown in Table 4. 

### 6.1. Kenaf (Hibiscus Cannabinus L.) Seeds

Kenaf (*Hibiscus cannabinus* L.) is a tall yearly herbaceous and woody tropical plant. It has attracted considerable attention as a multi-purpose plant with great potential for fiber, energy, and feedstock [130]. Kenaf originated in Africa, and disseminated in the 1900s in Asia (India and China) and the 1940s from Asia to central and northern USA. Nowadays, its cultivation is in many tropics and subtropic countries worldwide [130,131]. 

Kenaf seeds are the primary by-product of the kenaf plant as they are usually disposed of after harvesting [125] and only 2% of the total 1000 kg/hectare seed yields are required for a hectare of kenaf plantation [132]. Kenaf seeds contain essential nutrients, e.g., fiber, oil, proteins, and antioxidants, and might play important roles in value-added plant-based food products [133]. In particular, kenaf seed contained 24.93% crude protein, 18.94% fat, 13.45% fiber, 5.01% moisture, 4.50% ash, 33.10% carbohydrate, 1.30% flavonoid, and 381.00 mg Gallic Acid Equivalent (GAE)/g total phenolic content and a considerable amount of Na, Ca, Mg, P, and Fe minerals [134]. Previous research reported that kenaf seed contains 30–33% proteins, and 16–22% oil and primary fatty acids in kenaf seed oil are palmitic, oleic, and linoleic acids [135,136,137].

Kenaf seed oil was recently introduced as a new source of functional edible oil, with anti-cancer properties [138,139,140,141,142] and antioxidant activity [143]. A large amount of defatted kenaf seed meals (DKSM) will be generated due to kenaf seed oil extraction. In research, the protein content for DKSM was 26.19% [125], and the importance of DKSM as an alternative source of dietary protein has been shown. DKSM has been further processed into protein concentrates with good functional properties and suitable for food products as functional ingredients [144,145]. DKSM is also suggested as an effective secondary antioxidant to prevent hydroperoxide decomposition [125]. Hence, it can be used as a food preservative, as a functional food component, and as a nutraceutical formulation because of its potent antioxidant properties [125]. Interestingly, no study has been reported on the DF of DKSM.

### 6.2. Hemp (Cannabis Sativa L.) Seeds

The historical utilization of *Cannabis sativa* L. has been debatable. It can be categorized into two types, drug (marijuana) and non-drug (hemp). The drug type is commonly aimed for remedial and recreational purposes, while the non-drug type is essential to food and fiber applications. Over the last two decades, there has been renewed attention to hemp seed due to its nutritional and pharmaceutical potential. The cultivation of hemp seed at low tetrahydrocannabinol (THC) levels (<0.3% *w*/*w*) have been legalized in Canada, Australia, and most recently, the United States [128]. Hemp seeds contain approximately 6–7% moisture, 25–30% protein, 25–30% oil, and 30–40% fiber [146]. However, this percentage differs widely among various hemp cultivars [128]. For commercial use, *Finola* cultivar is the commonly planted hemp among more than 40 cultivars that have been reported [147]. Consequently, various chemical proportions of hemp seed cultivars provide a wide variation of hemp-added food products in terms of physicochemical, nutritional, and sensory properties [148]. 

The high content of unsaturated fatty acid (>90%) and a desirable balance of ω-6 and ω-3 fatty acids has led to a great demand for hemp seed. Cold-pressing and solvent extraction are some of developed methods for hemp seed oil extraction [149]. A by-product of cold-pressing hemp oil processes, called oil press cake, is rich in protein, fiber, minerals, phytochemicals, omega-6 (linoleic), omega-3 (alpha-linolenic) and gamma-tocopherols [126]. Albumin and globulin, which are characterized by their exceptionally high level of glutamic acid and arginine, become the main constituents composed of hemp seed protein [128]. These two types of protein showed a positive effect in modulating organ function and human metabolism [150]. Furthermore, hempseed protein equals egg white and soy protein [151]. Hemp proteins are usually well-tolerated and have occasional or mild allergic reactions. According to Mamone et al. [152], hemp seed protein isolate prepared by a general soy protein processing method produced no recognized allergens.

Moreover, some antioxidative bioactive peptides have been isolated from hemp seed protein [153]. In contrast, hemp seeds contain some anti-nutrients, most remarkably phytic acid and trypsin inhibitors [154]. Owing to the contradictory effects of these compounds on the human digestive system, their utilization in the food system has been controversial [155,156]. Hemp seed is also rich in polyphenols, primarily hydroxycinnamic and lignanamide. Several chemical and in vitro experiments show the anti-radical potential of these compounds [157,158,159].

Hemp seed contains both water-insoluble and water-soluble fiber at a ratio of about 80:20 [151], which is comparable to other legumes and seeds such as lupin seed and flaxseed [160]. Only a small amount of fiber resides in the cotyledon part of the seed, with the highest from the seed hull [128]. Furthermore, the dehulling process of seed can eliminate three-fourth of the seed fiber portion as most of the carbohydrates exist in the outer layer of hemp seed [146]. Vonapartis et al. [148] reported the insoluble DF of hemp seed consisted of cellulose, lignin, and hemicellulose for approximately 46%, 31% and 22%, respectively. However, literature related to the functional properties of hemp seed fiber is currently unavailable.

### 6.3. Sesame (Sesamum Indicum L.) Seeds

Sesame (*Sesamum indicum* L.) has become one of mankind’s oldest and important oilseeds. It is commonly used in confectionery and bakery products. They are rich sources of fat, protein, carbohydrate, DF, magnesium, zinc, and other minerals [161]. The extraction of sesame oil results in the production of semi-defatted sesame cake (SDSC) with protein (50%), crude fiber (10.8 g/100 g) and calcium (1.5 g/100 g) contents [162,163]. Generally, this nutritious defatted cake has been used as feed for fish, swine, and poultry, industries or disposed as waste. Discarding these products typically implies an issue further compounded by legal restrictions [129]. Innovative facets about using these residues as co-products for more value-added ingredients, food additives or supplements with high nutritional potential are thus gaining interest among researchers. Their recovery and usage are ecologically and economically desirable [163].

## 7. Conclusions

This review article highlights the fractionation method of DF, which is essential for the isolation and quantification of its constituents. Furthermore, the characteristics of DF fractions may explain its functionality in food products and provide clues to their physiological potential with extension to industrial utilizations. The growing production of edible oil from seeds opens the opportunity of potential sources of DF. Hence, researchers and food industries can explore the fractionation and characterization of DF from oilseed by-products to develop a value-added ingredient in the food and pharmaceutical industries. 

## Figures and Tables

**Table 1:** Analytical techniques for structural characterization of dietary fiber (DF).

Polysaccharides	Analysis of Structural Characterization	References
Alginates from brown seaweeds and carrageenans from red seaweeds	NMR, FTIR and SEC	[40]
Soluble DF from black soybean hulls	Monosaccharides composition, molecular weight, FTIR, SEM	[45]
Nettle seed gum	FTIR analysis and monosaccharide composition	[46]
Soluble DF from wheat bran	Molecular weight (SEC-MALLS), monosaccharide composition, FTIR, SEM	[47]
Galactomannan from *Prosopis ruscifolia* seeds	Monosaccharide composition (GC-MS), structure (NMR) and viscosity molecular weight (Huggins plot)	[48]
Indigestible carbohydrates from wheat processing	AEC, SEC, NMR	[49]
Flaxseed gum	Molecular weight distribution, monosaccharide composition (HPLC), FTIR, NMR	[50]
Cell wall polysaccharides from beans, lentils, peas and chickpeas	Monosaccharides composition (HPLC), molecular weight (chromatographic), galacturonic acid measurement (colorimetric method), microstructure (brightfield microscopy)	[13]
Insoluble DFs from Sichuan natural fermented pickle vegetables	Infrared spectroscopy, TG, SEM	[51]
Galactomannans from *Prosopis affinis*	HPLC, GC-MS, NMR spectroscopy, intrinsic viscosity and viscosity-average molecular weight	[52]
Resistant starch from Laird lentils (*Lens culinaris*) seeds	SEC, NMR, FTIR, SEM	[53]
Galactomannans from *Adenanthera pavonina, Caesalpinia pulcherrima, Gleditsia triacanthos* and *Sophora japonica* seeds	Monosaccharides composition, intrinsic viscosity, viscosity average molecular mass	[54]
Polysaccharide from *Lycium barbarum* L. fruit	FTIR, GC–MS, NMR	[29]
High DF powder from lime residues	SEM	[55]
Soluble DF from potato pulp	Monosaccharide content, molecular weight distribution	[56]

NMR, nuclear magnetic resonance; FTIR, Fourier-transform infrared spectroscopy; AEC, anion-exchange chromatography; SEC, size-exclusion chromatography; SEM, scanning electron microscope, GC-MS, gas chromatography–mass spectrometry; TG, thermogravimetry; HPLC, high-performance liquid chromatography.

**Table 2:** Functional and physicochemical characterization of DFs.

Sources	Analysis of Functional and Physicochemical	References
Galactomannans from mesquite seeds (*Prosopis* spp.)	Solubility, EC, ES	[81]
DF from sesame seed coats (testae)	WHC, OHC, bulk density, antioxidant activities	[82]
Soluble DFs from black soybean hulls	WHC, WSC, and OHC	[45]
Soluble DFs from wheat bran	Thermal properties (DSC), antioxidant activities	[47]
Flaxseed gum	Antioxidant activities	[50]
DFs derived from defatted rice bran	Viscosity	[79]
Insoluble DFs from Sichuan natural fermented pickle vegetables	WHC, OHC, swelling, antioxidant activity, polyphenol content, bile-acid salts adsorption capacities, and heavy metal adsorption.	[51]
High DF powder from lime residues	Health-related functional properties (GAC, GRI and BRI)	[55]

EC, emulsion capacity; ES, emulsion stability; WHC, water-holding capacity; OHC, oil-holding capacity; WSC, water-swelling capacity; DSC, differential scanning calorimetry; GAC, glucose adsorption capacity; GRI, glucose retardation index; BRI, bile-acid retardation index.

**Table 3:** Rheological characterization of DFs.

Samples	Rheological Measurement	References
Soluble DFs from wheat bran	Steady shear tests, dynamic oscillatory	[47]
Soluble and insoluble DFs from oat, pea, apple, lemon	Flow measurement, dynamic oscillatory, frequency sweep tests	[117]
Soluble DF from potato pulp	Steady flow tests	[56]
Insoluble date fiber incorporated wheat flour dough	Oscillatory rheological measurements, frequency sweep tests	[118]
Galactomannan from *Prosopis ruscifolia* seeds	Shear continuous and oscillatory assays	[48]
Wheat bran DF	Dynamic rheological properties	[119]
*Camelina sativa* gum	Apparent viscosity measurement, dynamic viscoelastic measurement frequency	[104]
Galactomannan extracted from *Trigonella foenum-graecum* (Fenugreek) Seed	Steady shear flow and dynamic rheology measurements	[120]
Mucilage extracted from *Opuntia ficus indica* (L. Miller)	Steady-shear flow and steady oscillatory flow measurements	[121]
Cell wall polysaccharides from beans, lentils, peas and chickpeas	Viscosity	[13]
Flour from green bananas	Flow properties, viscoelastic properties	[11]
*Amaranthus quitensis* and *Amaranthus caudatus*	Viscosity, viscoelastic properties	[122]
Sesame seeds and by-products	Viscosity	[123]
Quinoa seeds	Dynamic oscillatory rheology, viscoelasticity	[110]

**Table 4:** Nutritional composition of defatted seeds.

**Defatted** **Seeds**	**Nutritional Composition (%)**					**References**
	**Moisture**	**Ash**	**Protein**	**Fat**	**Carbohydrate**	
Kenaf	9.34	6.65	26.19	0.73	57.09	[125]
Hemp	6–7	5.24–7.08	32.21–40.7	2.1–10.2	30.5–46.67	[126,127,128]
Sesame	7.12–7.57	3.41–3.62	45.31–45.88	13.31–14.52	18.7–20.08	[129]

## Data Availability

Not applicable.

## References

[1] Hipsley E.H. (1953). Dietary “Fibre” and Pregnancy Toxaemia. BMJ.

[2] Valiente C., Mollá E., Martín-Cabrejas M.M., López-Andréu F.J., Esteban R.M. (1996). Cadmium Binding Capacity of Cocoa and Isolated Total Dietary Fibre under Physiological pH Conditions. J. Sci. Food Agric..

[3] Schneeman B.O. (2008). Dietary Fibre and Gastrointestinal Function. Advanced Dietary Fibre Technology.

[4] Trowell H., Southgate D., Wolever T., Leeds A., Gassull M., Jenkins D. (1976). Dietary Fibre Redefined. Lancet.

[5] Hussain S., Jõudu I., Bhat R. (2020). Dietary fiber from underutilized plant resources-A positive approach for valorization of fruit and vegetable wastes. Sustainability.

[6] Asp N.-G. (1987). Definition and analysis of dietary fibre. Scand. J. Gastroenterol..

[7] (1989). Recommended Dietary Allowances.

[8] Gupta P., Premavalli K.S. (2011). In-Vitro Studies on Functional Properties of Selected Natural Dietary Fibers. Int. J. Food Prop..

[9] Fleury N., Lahaye M. (1991). Chemical and physico-chemical characterisation of fibres from Laminaria digitata (kombu breton): A physiological approach. J. Sci. Food Agric..

[10] Gourgue C., Champ M., Guillon F., Delort-Laval J. (1994). Effect of Extrusion-Cooking on the Hypoglycaemic Properties of Citrus Fibre: An In-Vitro Study. J. Sci. Food Agric..

[11] Segundo C., Román L., Gómez M., Martínez M.M. (2017). Mechanically fractionated flour isolated from green bananas (M. cavendishii var. nanica) as a tool to increase the dietary fiber and phytochemical bioactivity of layer and sponge cakes. Food Chem..

[12] Verspreet J., Damen B., Broekaert W.F., Verbeke K., Delcour J.A., Courtin C.M. (2016). A Critical Look at Prebiotics Within the Dietary Fiber Concept. Annu. Rev. Food Sci. Technol..

[13] Brummer Y., Kaviani M., Tosh S.M. (2015). Structural and functional characteristics of dietary fibre in beans, lentils, peas and chickpeas. Food Res. Int..

[14] Malcolmson L., Han J. (2019). Pulse Processing and Utilization of Pulse Ingredients in Foods. Health Benefits of Pulses.

[15] Soukoulis C., Aprea E. (2012). Cereal Bran Fractionation: Processing Techniques for the Recovery of Functional Components and their Applications to the Food Industry. Recent Pat. Food Nutr. Agric..

[16] Elleuch M., Bedigian D., Roiseux O., Besbes S., Blecker C., Attia H. (2011). Dietary fibre and fibre-rich by-products of food processing: Characterisation, technological functionality and commercial applications: A review. Food Chem..

[17] Southgate D.A.T. (1969). Determination of carbohydrates in foods II.—Unavailable carbohydrates. J. Sci. Food Agric..

[18] Southgate D.A.T. (2009). The Definiton and Analysis of Dietary Fibre. Nutr. Rev..

[19] Anderson N.E., Ydesdale F.M.C. (1980). An Analysis of the Dietary Fiber, Content of a Standard Wheat Bran. J. Food Sci..

[20] Monte W.C., Maga J.A. (1980). Extraction and isolation of soluble and insoluble fiber fractions from the pinto bean (*Phaseolus vulgaris*). J. Agric. Food Chem..

[21] Graham H., Groen Rydberg M.B., Aaman P. (1988). Extraction of soluble dietary fiber. J. Agric. Food Chem..

[22] Czuchajowska Z., Pomeranz Y. (1994). Process for Fractionating Legumes to Obtain Pure Starch and a Protein Concentrate. U.S. Patent.

[23] Otto T., Baik B.-K., Czuchajowska Z. (1997). Wet Fractionation of Garbanzo Bean and Pea Flours. Cereal Chem. J..

[24] Wang J., Suo G., De Wit M., Boom R.M., Schutyser M.A.I. (2016). Dietary fibre enrichment from defatted rice bran by dry fractionation. J. Food Eng..

[25] Zijlstra R.T., van Kessel A.G., Drew M.D. (2004). Ingredient Fractionation: The Value of Value-Added Processing for Animal Nutrition “The Worth of the Sum of Parts versus the Whole ”. Proceedings of the 25th Western Nutrition Conference.

[26] Yáñez J.L., Beltranena E., Zijlstra R.T. (2014). Dry fractionation creates fractions of wheat distillers dried grains and solubles with highly digestible nutrient content for grower pigs1. J. Anim. Sci..

[27] Zhang X., Beltranena E., Christensen C., Yu P. (2012). Use of a Dry Fractionation Process to Manipulate the Chemical Profile and Nutrient Supply of a Coproduct from Bioethanol Processing. J. Agric. Food Chem..

[28] Mukhiddinov Z. (2000). Isolation and structural characterization of a pectin homo and ramnogalacturonan. Talanta.

[29] Zou S., Zhang X., Yao W., Niu Y., Gao X. (2010). Structure characterization and hypoglycemic activity of a polysaccharide isolated from the fruit of *Lycium barbarum* L.. Carbohydr. Polym..

[30] Alba K., MacNaughtan W., Laws A.P., Foster T.J., Campbell G.M., Kontogiorgos V. (2018). Fractionation and characterisation of dietary fibre from blackcurrant pomace. Food Hydrocoll..

[31] TYLER R.T. (1984). Impact Milling Quality of Grain Legumes. J. Food Sci..

[32] Farooq Z., Boye J.I. (2011). Novel food and industrial applications of pulse flours and fractions. Pulse Foods.

[33] Azarpazhooh E., Boye J.I. (2012). Composition of Processed Dry Beans and Pulses. Dry Beans and Pulses Production, Processing and Nutrition.

[34] Tosh S.M., Yada S. (2010). Dietary fibres in pulse seeds and fractions: Characterization, functional attributes, and applications. Food Res. Int..

[35] Azizi M.N., Loh T.C., Foo H.L., Teik Chung E.L. (2021). Is Palm Kernel Cake a Suitable Alternative Feed Ingredient for Poultry?. Animals.

[36] Morris E.R. (2008). Assembly and Rheology of Non-Starch Polysaccharides. Advanced Dietary Fibre Technology.

[37] Garcia-Vaquero M. (2019). Analytical Methods and Advances to Evaluate Dietary Fiber. Dietary Fiber: Properties, Recovery, and Applications.

[38] Jonsson H. (2010). Exploring the Structure of Oligo- and Polysaccharides Synthesis and NMR Spectroscopy Studies. Ph.D. Thesis.

[39] Bai Y., Shi Y.-C. (2016). Chemical structures in pyrodextrin determined by nuclear magnetic resonance spectroscopy. Carbohydr. Polym..

[40] Youssouf L., Lallemand L., Giraud P., Soulé F., Bhaw-Luximon A., Meilhac O., D’Hellencourt C.L., Jhurry D., Couprie J. (2017). Ultrasound-assisted extraction and structural characterization by NMR of alginates and carrageenans from seaweeds. Carbohydr. Polym..

[41] Jiménez-Escrig A., Sánchez-Muniz F.J. (2000). Dietary fibre from edible seaweeds: Chemical structure, physicochemical properties and effects on cholesterol metabolism. Nutr. Res..

[42] Roehrig K.L. (1988). The physiological effects of dietary fiber—A review. Food Hydrocoll..

[43] Abdul-Hamid A., Luan Y.S. (2000). Functional properties of dietary fibre prepared from defatted rice bran. Food Chem..

[44] Olson A., Gray G.M., Chiu M. (1987). Chemistry and analysis of soluble dietary fiber. Food Technol..

[45] Shen M., Weihao W., Cao L. (2020). Soluble dietary fibers from black soybean hulls: Physical and enzymatic modification, structure, physical properties, and cholesterol binding capacity. J. Food Sci..

[46] Kutlu G., Bozkurt F., Tornuk F. (2020). Extraction of a novel water-soluble gum from nettle (Urtica dioica) seeds: Optimization and characterization. Int. J. Biol. Macromol..

[47] Yan J.-K., Wu L.-X., Cai W.-D., Xiao G.-S., Duan Y., Zhang H. (2019). Subcritical water extraction-based methods affect the physicochemical and functional properties of soluble dietary fibers from wheat bran. Food Chem..

[48] Busch V.M., Kolender A.A., Santagapita P.R., Buera M.P. (2015). Vinal gum, a galactomannan from Prosopis ruscifolia seeds: Physicochemical characterization. Food Hydrocoll..

[49] Haskå L., Nyman M., Andersson R. (2010). Characterization of indigestible carbohydrates in various fractions from wheat processing. Cereal Chem..

[50] Safdar B., Pang Z., Liu X., Jatoi M.A., Mehmood A., Rashid M.T., Ali N., Naveed M. (2019). Flaxseed gum: Extraction, bioactive composition, structural characterization, and its potential antioxidant activity. J. Food Biochem..

[51] Jin Q., Xie F., Luo J., Huang X., Wen J., Zhang W., Wu J., He J., Wang Z. (2018). Investigation of Functional and Structural Properties of Insoluble Dietary Fiber From Sichuan Natural Fermented Pickles With Different Salting Treatments. Starch Stärke.

[52] Vilaró P., Bennadji Z., Budelli E., Moyna G., Panizzolo L., Ferreira F. (2018). Isolation and characterization of galactomannans from Prosopis affinis as potential gum substitutes. Food Hydrocoll..

[53] Ma Z., Yin X., Hu X., Li X., Liu L., Boye J.I. (2018). Structural characterization of resistant starch isolated from Laird lentils (Lens culinaris) seeds subjected to different processing treatments. Food Chem..

[54] Cerqueira M.A., Pinheiro A.C., Souza B.W.S., Lima Á.M.P., Ribeiro C., Miranda C., Teixeira J.A., Moreira R.A., Coimbra M.A., Gonçalves M.P. (2009). Extraction, purification and characterization of galactomannans from non-traditional sources. Carbohydr. Polym..

[55] Peerajit P., Chiewchan N., Devahastin S. (2012). Effects of pretreatment methods on health-related functional properties of high dietary fibre powder from lime residues. Food Chem..

[56] Cheng L., Zhang X., Hong Y., Li Z., Li C., Gu Z. (2017). Characterisation of physicochemical and functional properties of soluble dietary fibre from potato pulp obtained by enzyme-assisted extraction. Int. J. Biol. Macromol..

[57] BeMiller J.N. (2019). Monosaccharides. Carbohydrate Chemistry for Food Scientists.

[58] Vaclavik V.A., Christian E.W. (2014). Food Science Text Series Essentials of Food Science.

[59] Nielsen S.S. (2017). Food Analysis.

[60] Yan X., Ye R., Chen Y. (2015). Blasting extrusion processing: The increase of soluble dietary fiber content and extraction of soluble-fiber polysaccharides from wheat bran. Food Chem..

[61] Yadav M.P., Parris N., Johnston D.B., Hicks K.B. (2008). Fractionation, characterization, and study of the emulsifying properties of corn fiber gum. J. Agric. Food Chem..

[62] BeMiller J.N. (2019). Polysaccharides. Carbohydrate Chemistry for Food Scientists.

[63] Swadesh J.K., Swadesh J.K. (2000). HPLC.

[64] Valand R., Tanna S., Lawson G., Bengtström L. (2020). A review of Fourier Transform Infrared (FTIR) spectroscopy used in food adulteration and authenticity investigations. Food Addit. Contam. Part A.

[65] Munajad A., Subroto C., Suwarno (2018). Fourier Transform Infrared (FTIR) Spectroscopy Analysis of Transformer Paper in Mineral Oil-Paper Composite Insulation under Accelerated Thermal Aging. Energies.

[66] Cui H., Abu-Siada A., Li S., Islam S. Correlation between dissolved gases and oil spectral response. Proceedings of the 2017 1st International Conference on Electrical Materials and Power Equipment (ICEMPE).

[67] Stuart B.H. (2004). Infrared Spectroscopy: Fundamentals and Applications.

[68] Albert S., Albert K.K., Quack M. (2011). High-Resolution Fourier Transform Infrared Spectroscopy. Handbook of High-Resolution Spectroscopy.

[69] Ordoudi S.A., Cagliani L.R., Melidou D., Tsimidou M.Z., Consonni R. (2017). Uncovering a challenging case of adulterated commercial saffron. Food Control.

[70] Reis N., Botelho B.G., Franca A.S., Oliveira L.S. (2017). Simultaneous Detection of Multiple Adulterants in Ground Roasted Coffee by ATR-FTIR Spectroscopy and Data Fusion. Food Anal. Methods.

[71] Purohit S.R., Jayachandran L.E., Raj A.S., Nayak D., Rao P.S. (2019). X-ray diffraction for food quality evaluation. Evaluation Technologies for Food Quality.

[72] Leng Y. (2013). 2 X-Ray Diffraction Methods. Materials Characterization: Introduction to Microscopic and Spectroscopic Methods.

[73] Havlíček V., SpíŽek J., Havlíček V., Spížek J. (2014). Natural Products Analysis.

[74] Sahu J. (2014). Advances in Food Process Engineering. Introduction to Advanced Food Process Engineering.

[75] Leng Y. (2013). Scanning Electron Microscopy. Materials Characterization: Introduction to Microscopic and Spectroscopic Methods.

[76] Oikonomopoulou V.P., Krokida M.K., Karathanos V.T. (2011). Structural properties of freeze-dried rice. J. Food Eng..

[77] Fernández P.P., Martino M.N., Zaritzky N.E., Guignon B., Sanz P.D. (2007). Effects of locust bean, xanthan and guar gums on the ice crystals of a sucrose solution frozen at high pressure. Food Hydrocoll..

[78] Chaisawang M., Suphantharika M. (2006). Pasting and rheological properties of native and anionic tapioca starches as modified by guar gum and xanthan gum. Food Hydrocoll..

[79] Daou C., Zhang H. (2014). Functional and physiological properties of total, soluble, and insoluble dietary fibres derived from defatted rice bran. J. Food Sci. Technol..

[80] Blackwood A.D., Salter J., Dettmar P.W., Chaplin M.F. (2000). Dietary fibre, physicochemical properties and their relationship to health. J. R. Soc. Promot. Health.

[81] López-Franco Y.L., Cervantes-Montaño C.I., Martínez-Robinson K.G., Lizardi-Mendoza J., Robles-Ozuna L.E. (2013). Physicochemical characterization and functional properties of galactomannans from mesquite seeds (Prosopis spp.). Food Hydrocoll..

[82] Elleuch M., Bedigian D., Besbes S., Blecker C., Attia H. (2012). Dietary Fibre Characteristics and Antioxidant Activity of Sesame Seed Coats (Testae). Int. J. Food Prop..

[83] Niknam R., Ghanbarzadeh B., Ayaseh A., Rezagholi F. (2018). The effects of Plantago major seed gum on steady and dynamic oscillatory shear rheology of sunflower oil-in-water emulsions. J. Texture Stud..

[84] Chang Y.H., Cui S.W., Roberts K.T., Ng P.K.W., Wang Q. (2011). Evaluation of extrusion-modified fenugreek gum. Food Hydrocoll..

[85] Maskus H., Bourré L., Fraser S., Sarkar A., Malcolmson L. (2016). Effects of Grinding Method on the Compositional, Physical, and Functional Properties of Whole and Split Yellow Pea Flours. Cereal Foods World.

[86] Granizo D.P., Reuhs B.L., Stroshine R., Mauer L.J. (2007). Evaluating the solubility of powdered food ingredients using dynamic nuclear magnetic resonance (NMR) relaxometry. LWT—Food Sci. Technol..

[87] Mirhosseini H., Amid B.T. (2013). Effect of different drying techniques on flowability characteristics and chemical properties of natural carbohydrate-protein Gum from durian fruit seed. Chem. Cent. J..

[88] Kunzek H., Müller S., Vetter S., Godeck R. (2002). The significance of physico chemical properties of plant cell wall materials for the development of innovative food products. Eur. Food Res. Technol..

[89] Robertson J.A., de Monredon F.D., Dysseler P., Guillon F., Amado R., Thibault J.-F. (2000). Hydration Properties of Dietary Fibre and Resistant Starch: A European Collaborative Study. LWT—Food Sci. Technol..

[90] Raghavendra S.N., Rastogi N.K., Raghavarao K.S.M.S., Tharanathan R.N. (2004). Dietary fiber from coconut residue: Effects of different treatments and particle size on the hydration properties. Eur. Food Res. Technol..

[91] Chinma C.E., Ramakrishnan Y., Ilowefah M., Hanis-Syazwani M., Muhammad K. (2015). REVIEW: Properties of Cereal Brans: A Review. Cereal Chem. J..

[92] Esposito F., Arlotti G., Maria Bonifati A., Napolitano A., Vitale D., Fogliano V. (2005). Antioxidant activity and dietary fibre in durum wheat bran by-products. Food Res. Int..

[93] Tejada-Ortigoza V., García-Cayuela T., Welti-Chanes J., Cano M.P., Torres J.A., Welti-Chanes J., Serna-Saldívar S.O., Campanella O., Tejada-Ortigoza V. (2020). Emerging Technologies for the Extraction and Modification of Dietary Fiber. Science and Technology of Fibers in Food Systems.

[94] Tejada-Ortigoza V., Garcia-Amezquita L.E., Serna-Saldívar S.O., Welti-Chanes J. (2016). Advances in the Functional Characterization and Extraction Processes of Dietary Fiber. Food Eng. Rev..

[95] Grigelmo-Miguel N., Martín-Belloso O. (2000). The quality of peach jams stabilized with peach dietary fiber. Eur. Food Res. Technol..

[96] Zha X.-Q., Wang J.-H., Yang X.-F., Liang H., Zhao L.-L., Bao S.-H., Luo J.-P., Xu Y.-Y., Zhou B.-B. (2009). Antioxidant properties of polysaccharide fractions with different molecular mass extracted with hot-water from rice bran. Carbohydr. Polym..

[97] Guo W., Beta T. (2013). Phenolic acid composition and antioxidant potential of insoluble and soluble dietary fibre extracts derived from select whole-grain cereals. Food Res. Int..

[98] Quirós-Sauceda A.E., Palafox-Carlos H., Sáyago-Ayerdi S.G., Ayala-Zavala J.F., Bello-Perez L.A., Álvarez-Parrilla E., de la Rosa L.A., González-Córdova A.F., González-Aguilar G.A. (2014). Dietary fiber and phenolic compounds as functional ingredients: Interaction and possible effect after ingestion. Food Funct..

[99] Del Pino-García R., González-SanJosé M.L., Rivero-Pérez M.D., García-Lomillo J., Muñiz P. (2016). Total antioxidant capacity of new natural powdered seasonings after gastrointestinal and colonic digestion. Food Chem..

[100] Damodaran S., Parkin K.L., Fennema O.R., Parkin K.L., Fennema O.R. (2007). Fennema’s Food Chemistry.

[101] Gould W.A., Gould R.W. (2001). Water Activity. Total Quality Assurance for the Food Industries.

[102] Reid D.S. (2008). Water Activity: Fundamentals and Relationships. Water Activity in Foods.

[103] Figura L.O., Teixeira A.A., Figura L.O., Teixeira A.A. (2007). Food Physics.

[104] Sanchez Y.M. (2014). Characterization and Rheological Properties of Camelina Sativa Gum: Interactions with Xanthan Gum, Guar Gum, and Locust Bean Gum. Ph.D. Thesis.

[105] Tabilo-Munizaga G., Barbosa-Cánovas G.V. (2005). Rheology for the food industry. J. Food Eng..

[106] Marcotte M., Taherian Hoshahili A.R., Ramaswamy H.S. (2001). Rheological properties of selected hydrocolloids as a function of concentration and temperature. Food Res. Int..

[107] Nielsen S.S., Nielsen S.S. (2010). Food Analysis Laboratory Manual.

[108] Ahmed J., Ptaszek P., Basu S. (2017). Food Rheology: Scientific Development and Importance to Food Industry. Advances in Food Rheology and Its Applications.

[109] García-Abuín A., Gómez-Díaz D., Navaza J.M. (2011). Apparent viscosity model for hydrocolloids blends. Electron. J. Environ. Agric. Food Chem..

[110] Ahmed J., Thomas L., Arfat Y.A. (2019). Functional, rheological, microstructural and antioxidant properties of quinoa flour in dispersions as influenced by particle size. Food Res. Int..

[111] Tornberg E. (2017). Influence of Fibers and Particle Size Distribution on Food Rheology. Advances in Food Rheology and Its Applications.

[112] Sanderson G.R. (1981). Applications of Xanthan gum. Br. Polym. J..

[113] Grigelmo-Miguel N., Gorinstein S., Martın-Belloso O. (1999). Characterisation of peach dietary fibre concentrate as a food ingredient. Food Chem..

[114] Elleuch M., Besbes S., Roiseux O., Blecker C., Deroanne C., Drira N.-E., Attia H. (2008). Date flesh: Chemical composition and characteristics of the dietary fibre. Food Chem..

[115] Von Borries-Medrano E., Jaime-Fonseca M.R., Aguilar-Méndez M.Á. (2017). Starch-Galactomannans Mixtures: Rheological and Viscosity Behavior in Aqueous Systems for Food Modeling. Solubility of Polysaccharides.

[116] Ahmed J., Taher A., Mulla M.Z., Al-Hazza A., Luciano G. (2016). Effect of sieve particle size on functional, thermal, rheological and pasting properties of Indian and Turkish lentil flour. J. Food Eng..

[117] Aydogdu A., Sumnu G., Sahin S. (2018). Effects of addition of different fibers on rheological characteristics of cake batter and quality of cakes. J. Food Sci. Technol..

[118] Ahmed J., Almusallam A.S., Al-Salman F., AbdulRahman M.H., Al-Salem E. (2013). Rheological properties of water insoluble date fiber incorporated wheat flour dough. LWT—Food Sci. Technol..

[119] Liu N., Ma S., Li L., Wang X. (2019). Study on the effect of wheat bran dietary fiber on the rheological properties of dough. Grain Oil Sci. Technol..

[120] Niknam R., Mousavi M., Kiani H. (2020). New studies on the galactomannan extracted from *Trigonella foenum-graecum* (Fenugreek) seed: Effect of subsequent use of ultrasound and microwave on the physicochemical and rheological properties. Food Bioprocess Technol..

[121] Quinzio C., Ayunta C., Alancay M., de Mishima B.L., Iturriaga L. (2018). Physicochemical and rheological properties of mucilage extracted from *Opuntia ficus indica* (L. Miller). Comparative study with guar gum and xanthan gum. J. Food Meas. Charact..

[122] Cornejo F., Novillo G., Villacrés E., Rosell C.M. (2019). Evaluation of the physicochemical and nutritional changes in two amaranth species (*Amaranthus quitensis* and *Amaranthus caudatus*) after germination. Food Res. Int..

[123] Elleuch M., Besbes S., Roiseux O., Blecker C., Attia H. (2007). Quality characteristics of sesame seeds and by-products. Food Chem..

[124] Kaur D., Wani A.A., Singh D.P., Sogi D.S. (2011). Shelf-life enhancement of butter, ice-cream, and mayonnaise by addition of lycopene. Int. J. Food Prop..

[125] Chan K.W., Khong N.M.H., Iqbal S., Mansor S.M., Ismail M. (2013). Defatted kenaf seed meal (DKSM): Prospective edible flour from agricultural waste with high antioxidant activity. LWT—Food Sci. Technol..

[126] Radočaj O., Dimić E., Tsao R. (2014). Effects of hemp (*Cannabis sativa* L.) seed oil press-cake and decaffeinated green tea leaves (*Camellia sinensis*) on functional characteristics of gluten-free crackers. J. Food Sci..

[127] Pap N., Hamberg L., Pihlava J., Hellström J., Mattila P., Eurola M., Pihlanto A. (2020). Impact of enzymatic hydrolysis on the nutrients, phytochemicals and sensory properties of oil hemp seed cake (*Cannabis sativa* L. FINOLA variety). Food Chem..

[128] Leonard W., Zhang P., Ying D., Fang Z. (2020). Hempseed in food industry: Nutritional value, health benefits, and industrial applications. Compr. Rev. Food Sci. Food Saf..

[129] Prakash K., Naik S.N., Vadivel D., Hariprasad P., Gandhi D., Saravanadevi S. (2018). Utilization of defatted sesame cake in enhancing the nutritional and functional characteristics of biscuits. J. Food Process. Preserv..

[130] Mariod A.A., Saeed Mirghani M.E., Hussein I. (2017). Hibiscus cannabinus L. Kenaf. Unconventional Oilseeds and Oil Sources.

[131] Monti A., Monti A., Alexopoulou E. (2013). Kenaf: A Multi-Purpose Crop for Several Industrial Applications.

[132] Wei C.K. (2019). Cholesterol-lowering Properties of Defatted Kenaf Seed Meal and its Phenolics-Saponins-rich Extract in a Rat Model. Ph.D. Thesis.

[133] Giwa Ibrahim S., Karim R., Saari N., Wan Abdullah W.Z., Zawawi N., Ab Razak A.F., Hamim N.A., Umar R.A. (2019). Kenaf (*Hibiscus cannabinus* L.) seed and its potential food applications: A Review. J. Food Sci..

[134] Omenna E.C., Uzuegbu D.C., Okeleye D.D. (2017). Functional and nutritional properties of kenaf seed. EC Nutr..

[135] Hopkins C.Y., Chisholm M.J. (1959). Fatty acids of kenaf seed oil. J. Am. Oil Chem. Soc..

[136] Subbaram M.R., Rajagopal N.S., Paulose M.M., Subbarao R., Roomi M.W., Achaya K.T. (1964). Component fatty acid analyses by reversed-phase partition chromatography. J. Sci. Food Agric..

[137] Tolibaev I., Mukhamedova K.S., Glushenkova A.I., Tursunkhodzhaev A.S., Azizov T.B. (1986). Lipids of kenaf wastes. Chem. Nat. Compd..

[138] Yazan L.S., Abd Rahman N., Chan K.W., Wan Abd Ghani W.N.H., Tor Y.S., Foo J.B. (2016). Phenolics-saponins rich fraction of defatted kenaf seed meal exhibits cytotoxicity towards cancer cell lines. Asian Pac. J. Trop. Biomed..

[139] Chan K.W., Iqbal S., Khong N.M.H., Ooi D.-J., Ismail M. (2014). Antioxidant activity of phenolics–saponins rich fraction prepared from defatted kenaf seed meal. LWT—Food Sci. Technol..

[140] Foo J.B., Yazan L.S., Chan K.W., Tahir P.M., Ismail M. (2011). Kenaf seed oil from supercritical carbon dioxide fluid extraction induced G1 phase cell cycle arrest and apoptosis in leukemia cells. Afr. J. Biotechnol..

[141] Ghafar S.A.A., Yazan L.S., Tahir P.M., Ismail M. (2012). Kenaf seed supercritical fluid extract reduces aberrant crypt foci formation in azoxymethane-induced rats. Exp. Toxicol. Pathol..

[142] Yazan L.S., Foo J.B., Ghafar S.A.A., Chan K.W., Tahir P.M., Ismail M. (2011). Effect of kenaf seed oil from different ways of extraction towards ovarian cancer cells. Food Bioprod. Process..

[143] Chan K.W., Ismail M. (2009). Supercritical carbon dioxide fluid extraction of *Hibiscus cannabinus* L. seed oil: A potential solvent-free and high antioxidative edible oil. Food Chem..

[144] Ibrahim S.G., Mat Noh N.A., Wan Ibadullah W.Z., Saari N., Karim R. (2020). Water soaking temperature of kenaf (*Hibiscus cannabinus* L.) seed, coagulant types, and their concentrations affected the production of kenaf-based tofu. J. Food Process. Preserv..

[145] Mariod A.A., Fathy S.F., Ismail M. (2010). Preparation and characterisation of protein concentrates from defatted kenaf seed. Food Chem..

[146] House J.D., Neufeld J., Leson G. (2010). Evaluating the quality of protein from hemp seed (*Cannabis sativa* L.) products through the use of the protein digestibility-corrected amino acid score method. J. Agric. Food Chem..

[147] Schluttenhofer C., Yuan L. (2017). Challenges towards Revitalizing Hemp: A Multifaceted Crop. Trends Plant Sci..

[148] Vonapartis E., Aubin M.-P., Seguin P., Mustafa A.F., Charron J.-B. (2015). Seed composition of ten industrial hemp cultivars approved for production in Canada. J. Food Compos. Anal..

[149] Devi V., Khanam S. (2019). Study of ω-6 linoleic and ω-3 α-linolenic acids of hemp (*Cannabis sativa*) seed oil extracted by supercritical CO2 extraction: CCD optimization. J. Environ. Chem. Eng..

[150] Wu H., Wang Q., Ma T., Ren J. (2009). Comparative studies on the functional properties of various protein concentrate preparations of peanut protein. Food Res. Int..

[151] Callaway J.C. (2004). Hempseed as a nutritional resource: An overview. Euphytica.

[152] Mamone G., Picariello G., Ramondo A., Nicolai M.A., Ferranti P. (2019). Production, digestibility and allergenicity of hemp (*Cannabis sativa* L.) protein isolates. Food Res. Int..

[153] Malomo S.A., He R., Aluko R.E. (2014). Structural and functional properties of hemp seed protein products. J. Food Sci..

[154] Pojić M., Mišan A., Sakač M., Dapčević Hadnađev T., Šarić B., Milovanović I., Hadnađev M. (2014). Characterization of byproducts originating from hemp oil processing. J. Agric. Food Chem..

[155] Sánchez-Chino X., Jiménez-Martínez C., Dávila-Ortiz G., Álvarez-González I., Madrigal-Bujaidar E. (2015). Nutrient and non-nutrient components of legumes, and its chemopreventive activity: A Review. Nutr. Cancer.

[156] Thompson L.U. (1993). Potential health benefits and problems associated with antinutrients in foods. Food Res. Int..

[157] Chen T., He J., Zhang J., Li X., Zhang H., Hao J., Li L. (2012). The isolation and identification of two compounds with predominant radical scavenging activity in hempseed (seed of *Cannabis sativa* L.). Food Chem..

[158] Yan X., Tang J., Dos Santos Passos C., Nurisso A., Simoes-Pires C.A., Ji M., Lou H., Fan P. (2015). Characterization of lignanamides from hemp (*cannabis sativa* l.) seed and their antioxidant and acetylcholinesterase inhibitory activities. J. Agric. Food Chem..

[159] Zhou Y., Wang S., Ji J., Lou H., Fan P. (2018). Hemp (*Cannabis sativa* L.) Seed Phenylpropionamides Composition and Effects on Memory Dysfunction and Biomarkers of Neuroinflammation Induced by Lipopolysaccharide in Mice. ACS Omega.

[160] Mattila P., Mäkinen S., Eurola M., Jalava T., Pihlava J.-M., Hellström J., Pihlanto A. (2018). Nutritional value of commercial protein-rich plant products. Plant Foods Hum. Nutr..

[161] Prakash K., Naik S.N. (2014). Bioactive constituents as a potential agent in sesame for functional and nutritional application. J. Bioresour. Eng. Technol..

[162] Nascimento E.M. (2012). da G.C. do; Carvalho, C.W.P.; Takeiti, C.Y.; Freitas, D.D.G.C.; Ascheri, J.L.R. Use of sesame oil cake (*Sesamum indicum* L.) on corn expanded extrudates. Food Res. Int..

[163] Mohdaly A.A.A., Hassanien M.F.R., Mahmoud A., Sarhan M.A., Smetanska I. (2013). Phenolics Extracted from Potato, Sugar Beet, and Sesame Processing By-Products. Int. J. Food Prop..

